# Virtual screening of natural products as potential inhibitors of SARS-CoV-2 main protease, RNA-dependent RNA polymerase (RdRp) and Spike Protein: Database design, molecular docking and molecular dynamic study

**DOI:** 10.22038/AJP.2024.24271

**Published:** 2024

**Authors:** Motahareh Boozari, Zeinab Amiri Tehranizadeh, Hossein Hosseinzadeh

**Affiliations:** 1 *Department of Pharmacognosy, School of Pharmacy, Mashhad University of Medical Sciences, Mashhad, Iran *; 2 *Department of Medicinal Chemistry, School of Pharmacy, Mashhad University of Medical Sciences, Mashhad, Iran*; 3 *Department of Pharmacodynamics and Toxicology, School of Pharmacy, Mashhad University of Medical Sciences, Mashhad, Iran*; 4 *Pharmaceutical Research Center, Pharmaceutical Technology Institute, Mashhad University of Medical Sciences, Mashhad, Iran*

**Keywords:** COVID-19, Chebulagic acid, Fangchinoline, Suramin, Spike protein, RdRp

## Abstract

**Objective:**

COVID-19 is caused by the SARS-CoV-2 virus. In this study, around 300 herbal compounds were screened virtually to find the best anti-COVID-19 structures.

**Materials and Methods:**

An extensive search in electronic databases was done. Around 300 herbal compounds, which were previously proven to be antiviral structures, were extracted from articles and considered our primary database. Then, molecular docking studies were performed to find the best inhibitors of the main SARS-COV-2 proteins, including spike protein (PDB 7BWJ), RNA-dependent RNA polymerase (PDB 6M71) and main protease (PDB 5R7Z).

**Results:**

The molecular docking and dynamics studies revealed that fangchinoline as an alkaloid could bind to the main protease of the virus more potent than lopinavir (-42.26 vs. -30.9 kJ/mol). Fangchinoline can be orally active based on drug-like properties. According to the molecular dynamic study, the complex between the fangchinoline and SARS-CoV-2 main protease is stable. chebulagic acid is a benzopyrene tannin that could inhibit RNA-dependent RNA polymerase (RdRp) better than remdesivir (-43.9 vs. -28.8 kJ/mol). The molecular dynamic study showed that chebulagic acid-RdRp interaction is stable and strong. Furthermore, suramin could neutralize different variants of COVID-19 spike proteins (wild type, and alpha and beta variants). However, suramin is not orally active but it is a potential inhibitor for different coronavirus spike proteins.

**Conclusion:**

According to the promising *in silico* results of this study, fangchinoline, chebulagic acid and suramin could be introduced as potential lead compounds for COVID-19 treatment. We are hopeful to find a reliable remedy shortly through natural compounds.

## Introduction

COVID-19 is a life-threatening disease caused by the SARS-CoV-2 virus. This virus was initially known as the 2019 novel coronavirus (2019-nCoV) and the disease caused by it as the 2019 coronavirus disease (COVID-19) by the World Health Organization. Until January 2023, 6,831,146 deaths occurred due to COVID-19 infection. Identifying and developing a reliable treatment modality for patient care will be imperative in the near future. Herbal medicines have always been the center of attention for controlling respiratory diseases. Active constituents of herbal plants can interact efficiently with the proteins of the virus and neutralize the virus virulence. In this study, around 300 herbal compounds were screened virtually to find the best anti-COVID-19 structures. The previous coronavirus infection Severe Acute Respiratory Syndrome (SARS) occurred in 2003 in China and Middle East Respiratory Syndrome (MERS) in 2012 in Saudi Arabia (Drosten et al., 2003). During the first days of the coronavirus pandemic, many *in silico* screening studies on different SARS-CoV-2 targets based on the known databases were conducted, such as the ZINC database or other commercial databases (Singh and Florez, 2020; Yu et al., 2020; Ruan et al., 2020; Noureddine et al., 2021). Different studies have investigated the potential natural products for COVID-19 treatment (Boozari and Hosseinzadeh, 2021; Haidere et al., 2021; Brendler et al., 2021). Three important therapeutic strategies against COVID-19 are antiviral activity, anti‐inflammatory activity, and immunomodulatory effects. Based on clinical trials, the most important mechanisms of natural products are modulatory effects on the immune system, anti-inflammatory effects and antiviral activity (Babaei et al., 2021; Rameshrad et al., 2020).

In this study, we designed a small-scale database of effective anti-viral natural compounds and screened this database for the best compound against SARS-CoV-2 targets. Finally, molecular dynamic simulation was done to confirm the docking results.

**Figure 1:**
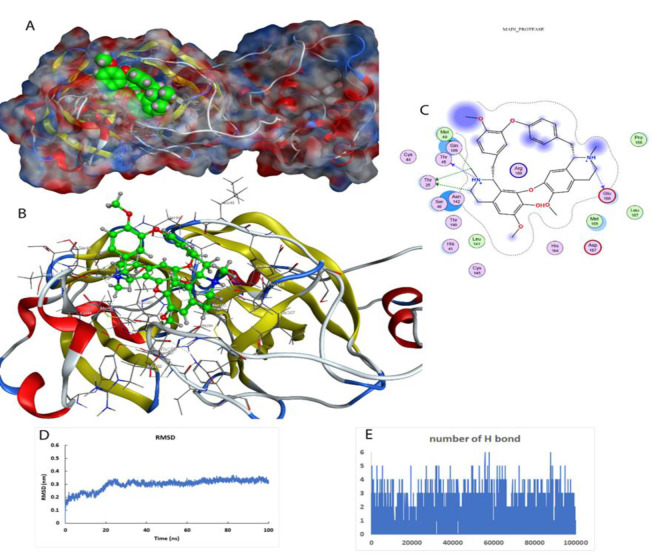
Interaction of fangchinoline with the main protease. A) The active site of the main protease and the position of fangchinoline are shown. The protein is shown as surface and ligand with spacer model in green color. B) The active site of the protein and the secondary structures of the protein around the ligand are shown (Ball-and-stick model). C) The two-dimensional model of the ligand position in the active site and the amino acids involved in creating an effective interaction are shown. D) Stability of the ligand-protein complex during 100 ns of simulation. E) The number of hydrogen bonds formed in the ligand-protein interaction during 100 ns of simulation.

**Figure 2:**
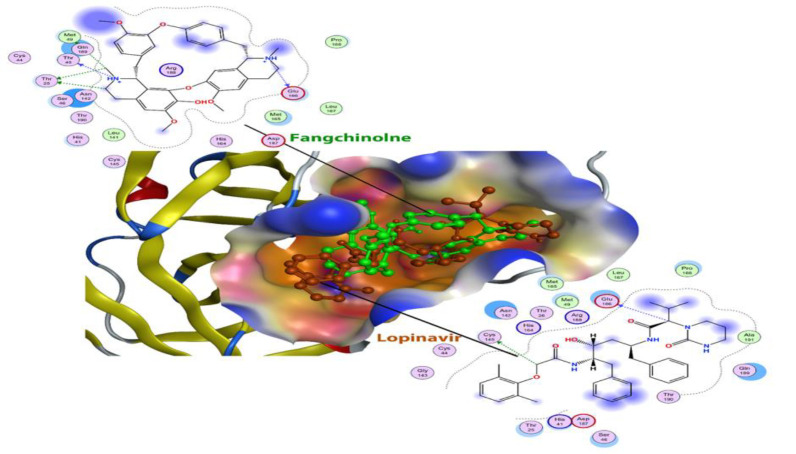
The two structures of lopinavir (brown) and fangsinoline (green) are shown in the active site of the main protease. The active site of the protein is shown based on the nature of the amino acids that are polar (red) or non-polar (orange).

**Figure 3:**
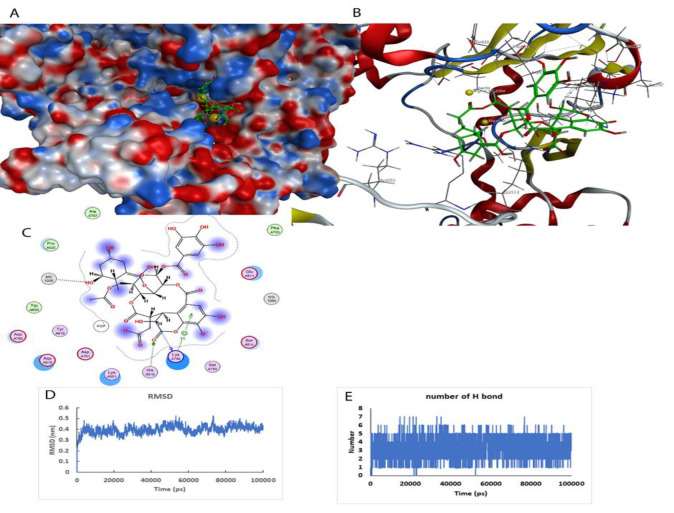
Chebulagic acid and RdRp interaction. A) The active site of the RdRp and the position chebulagic acid are shown. The protein is shown as surface and ligand with spacer model in green color. B) The active site of the protein and the secondary structures of the protein around the ligand are shown (Ball-and-stick model). C) The two-dimensional model of the ligand position in the active site and the amino acids involved in creating an effective interaction are shown. D) Stability of the ligand-protein complex during 100 ns of simulation. E) The number of hydrogen bonds formed in the ligand-protein interaction during 100 ns of simulation.

**Figure 4:**
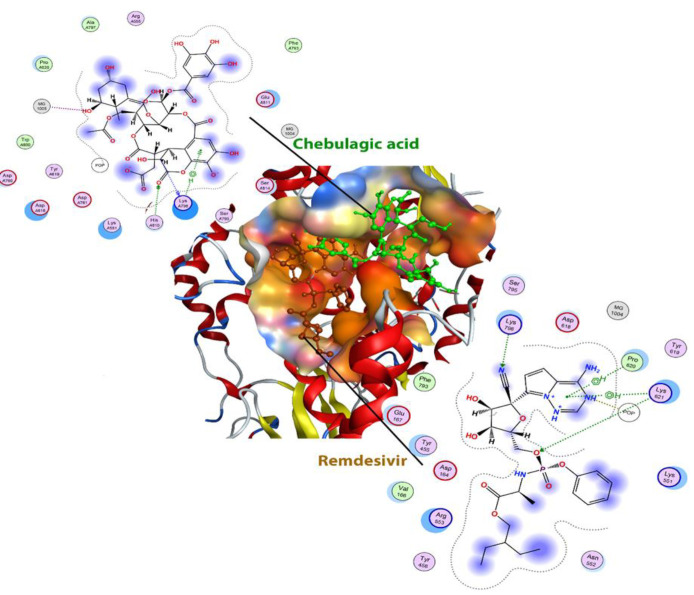
Structures of remdesivir (brown) and chebulagic acid (green) are shown in the active site of RdRp. The active site of the protein is shown based on the nature of the amino acids that are polar (red) or non-polar (orange).

**Figure 5:**
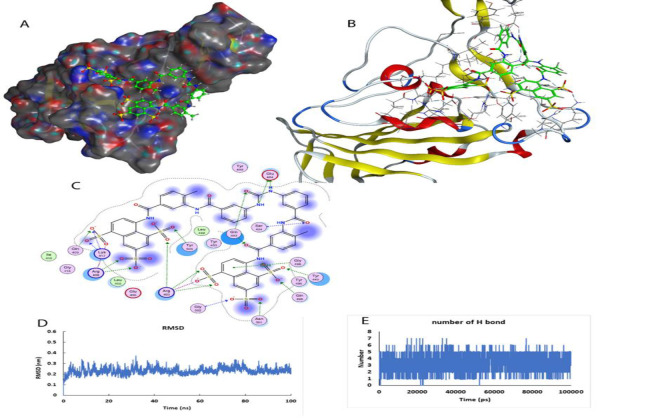
Interaction of suramin with Spike protein. A) The active site of spike protein and the suramin position are shown. The protein is shown as surface and ligand with spacer model in green color. B) The active site of the protein and the secondary structures of the protein around the ligand are shown (Ball-and-stick model). C) The two-dimensional model of the ligand position in the active site and the amino acids involved in creating an effective interaction are shown. D) Stability of the ligand-protein complex during 100 ns of simulation. E) The number of hydrogen bonds formed in the ligand-protein interaction during 100 ns of simulation

**Figure 6:**
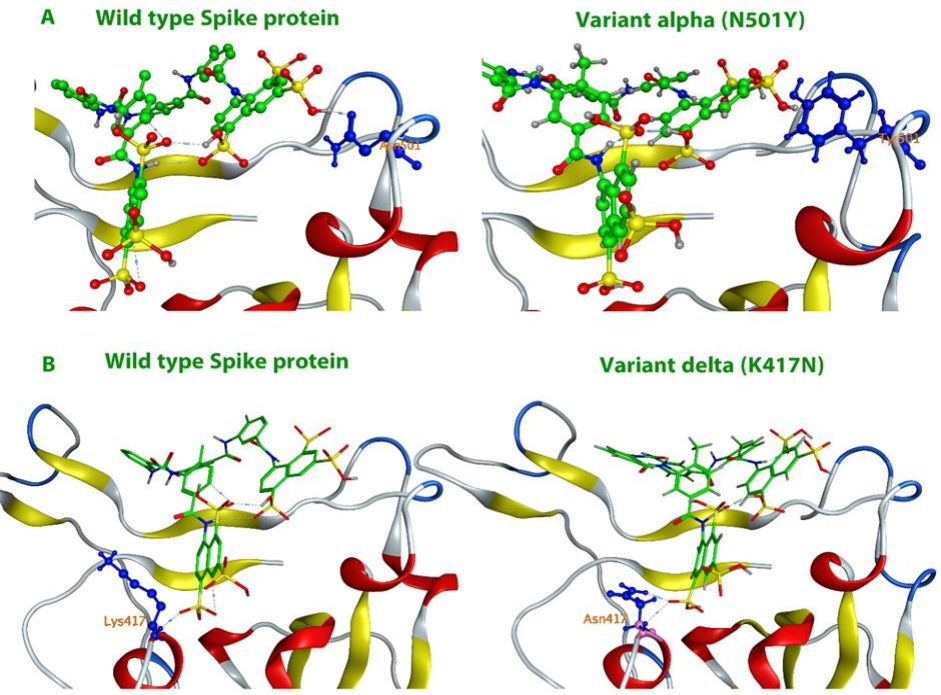
The active site of various mutant spike protein species and their interaction with suramin ligand (Ball-and-stick model). A) The changes related to the alpha mutation in the active site are shown and the changes in suramin binding to the active site (N501Y). B) The changes related to the delta mutation in the active site are shown and the changes in suramin binding to the active site (K417N).

**Table 1:** Natural products with inhibitory activity against SARS-CoV2 targets

Type	Compound name	Plant	Inhibitory activity	Ref.	
Abietane terpenoids	Rosmariquinone	*Salvia miltiorrhiza*	3CL^pro ^inhibitory activity IC_50_: 21.1 μM	(Park et al., 2012b)	
	Methyl tanshinonate	*Salvia miltiorrhiza*	3CL^pro ^inhibitory activity IC_50_: 21.1 μM	(Park et al., 2012b)	
	Dihydrotanshinone I	*Salvia miltiorrhiza*	3CL^pro ^inhibitory activity IC_50_: 14.4 μM	(Park et al., 2012b)	
			PL^pro^ inhibitory activity IC_50_: 4.9 μM		
	Tanshinone I	*Salvia miltiorrhiza*	PL^pro^ inhibitory activity IC_50_: 8.8 μM	(Park et al., 2012b)	
	Tanshinone IIA	*Salvia miltiorrhiza*	PL^pro^ inhibitory activity IC_50_: 1.6 μM	(Park et al., 2012b)	
	Cryptotanshinone	*Salvia miltiorrhiza*	PL^pro^ inhibitory activity IC_50_: 0.8 μM	(Park et al., 2012b)	
Quinone-methide triterpenes	Celastrol	*Triterygium regelii*	3CL^pro ^inhibitory activity IC_50_: 10.3 μM	(Ryu et al., 2010b)	
	Pristimerin	*Triterygium regelii*	3CL^pro ^inhibitory activity IC_50_: 5.5 μM	(Ryu et al., 2010b)	
	Tingenone	*Triterygium regelii*	3CL^pro ^inhibitory activity IC_50_: 9.9 μM	(Ryu et al., 2010b)	
	Iguesterin	*Triterygium regelii*	3CL^pro ^inhibitory activity IC_50_: 2.6 μM	(Ryu et al., 2010b)	
Triterpenoids	Betulinic acid	*Juniperus formosana*	3CL^pro ^inhibitory activity IC_50_: 10 μM	(Wen et al., 2007)	
Saponin	Glycyrrhizin	*Glycyrrhiza glabra*	SARS-associated virus replication inhibition	(Cinatl et al., 2003)	
Stilbenoid	Resveratrol	*Vitis vinifera*	Inhibited MERS-cov infection and decreased MERS-cov replication in vitro	(Lin et al., 2017)	
Flavonoids	Luteolin	Vegetable	Binds to the surface spike protein of SARS-cov (EC_50_ 10.6 μm)	(Yi et al., 2004)	
			3CL^pro ^inhibitory activity IC_50_: 20.2 μM	(Ryu et al., 2010a)	
	Hesperetin	*Isatis indigotica*	3CL^pro ^inhibitory activity IC_50_: 8.3 μM	(Lin et al., 2005)	
	Quercetin	Vegetable	3CL^pro ^inhibitory activity IC_50_: 23.8 μM	(Ryu et al., 2010a)	
	Amentoflavone	*Torreya nucifera*	3CL^pro ^inhibitory activity IC_50_: 8.3 μM	(Ryu et al., 2010a)	
	Tomentin A	*Paulownia tomentosa*	PL^pro^ inhibitory activity IC_50_: 6.2 μM	(Cho et al., 2013)	
	Tomentin B	*Paulownia tomentosa*	PL^pro^ inhibitory activity IC_50_: 6.1 μM	Cho et al., 2013)	
	Tomentin C	*Paulownia tomentosa*	PL^pro^ inhibitory activity IC_50_: 11.6 μM	Cho et al., 2013)	
	Tomentin D	*Paulownia tomentosa*	PL^pro^ inhibitory activity IC_50_: 12.5 μM	Cho et al., 2013)	
	Tomentin E	*Paulownia tomentosa*	PL^pro^ inhibitory activity IC_50_: 5.0 μM	Cho et al., 2013)	
	3'-O-methyldiplacol	*Paulownia tomentosa*	PL^pro^ inhibitory activity IC_50_: 9.5 μM	Cho et al., 2013)	
	4'-O-methyldiplacol	*Paulownia tomentosa*	PL^pro^ inhibitory activity IC_50_: 9.2 μM	Cho et al., 2013)	
	3'-O-methyldiplacone	*Paulownia tomentosa*	PL^pro^ inhibitory activity IC_50_: 13.2 μM	Cho et al., 2013)	
	4'-O-methyldiplacone	*Paulownia tomentosa*	PL^pro^ inhibitory activity IC_50_: 12.7 μM	Cho et al., 2013)	
	Mimulone	*Paulownia tomentosa*	PL^pro^ inhibitory activity IC_50_: 14.4 μM	Cho et al., 2013)	
	Diplacone	*Paulownia tomentosa*	PL^pro^ inhibitory activity IC_50_: 10.4 μM	Cho et al., 2013)	
	6- geranyl-4',5,7-trihydroxy-3',5'-dimethoxyflavanone	*Paulownia tomentosa*	PL^pro^ inhibitory activity IC_50_: 13.7 μM	Cho et al., 2013)	
	Bavachinin	*Psoralea corylifolia*	PL^pro^ inhibitory activity IC_50_: 38.4 μM		(Kim et al., 2014)
	Corylifol A	*Psoralea corylifolia*	PL^pro^ inhibitory activity IC_50_: 32.3 μM		(Kim et al., 2014)
	Isobavachalcone	*Psoralea corylifolia*	PL^pro^ inhibitory activity IC_50_: 18.3 μM		(Kim et al., 2014)
	4'-O-methylbavachalcone	*Psoralea corylifolia*	PL^pro^ inhibitory activity IC_50_: 10.1 μM		(Kim et al., 2014)
	Neobavaisoflavone	*Psoralea corylifolia*	PL^pro^ inhibitory activity IC_50_: 18.3 μM		(Kim et al., 2014)
	Psoralidin	*Psoralea corylifolia*	PL^pro^ inhibitory activity IC_50_: 4.2 μM		(Kim et al., 2014)
	Baicalin	*Scutellaria baicalensis*	Antiviral activity against SARS (EC_50_ 12.5 μg/ml)		(Chen et al., 2004)
	Xanthoangelol E	*Angelica keiskei*	3CL^pro ^inhibitory activity IC_50_: 11.4μM		(Park et al., 2016)
			PL^pro^ inhibitory activity IC_50_: 1.2 μM		
	Xanthoangelol B	*Angelica keiskei*	3CL^pro ^inhibitory activity IC_50_: 22.2 μM		(Park et al., 2016)
			PL^pro^ inhibitory activity IC_50_: 11.7 μM		
	xanthoangelol F	*Angelica keiskei*	PL^pro^ inhibitory activity IC_50_: 5.6 μM		(Park et al., 2016)
	xanthoangelol	*Angelica keiskei*	PL^pro^ inhibitory activity IC_50_: 11.7 μM		Park et al., 2016)
	Isobavachalcone	*Angelica keiskei*	PL^pro^ inhibitory activity IC_50_: 13.0 μM		Park et al., 2016)
Tannins	Theaflavin-3,3'digallate	*Camellia sinensis*	3CL^pro ^inhibitory activity IC_50_: 9.5 μM		(Lin et al., 2005)
	3-Isotheaflavin-3 gallate	*Camellia sinensis*	3CL^pro ^inhibitory activity IC_50_: 7 μM		(Lin et al., 2005)
	Tannic acid	*Camellia sinensis*	3CL^pro ^inhibitory activity IC_50_: 3 μM		(Lin et al., 2005)
Diarylheptanoids	Curcumin	*Curcuma longa*	3CL^pro ^inhibitory activity IC_50_: 40 μM		(Wen et al., 2007)
			PL^pro^ inhibitory activity IC_50_: 5.7 μM		(Park et al., 2012a)
	Hirsutenone	*Alnus japonica*	PL^pro^ inhibitory activity IC_50_: 4.1 μM		(Park et al., 2012a)
Alkaloids	lycorine	*Lycoris radiata*	HCoV-OC43 (EC50: 0.15 μM), MERS-CoV (EC50: 1.63μM) and HCoV-NL63 (EC50: 0.47μM).		(Shen et al., 2019)
	Emetine	*Carapichea ipecacuanha*	HCoV-OC43 (EC_50_: 0.30 μM), MERS-CoV (EC50: 0.34μM) and HCoV-NL63 (EC50: 1.43μM).		(Shen et al., 2019)
	Tylophorine	*Tylophora indica*	potent coronavirus replication inhibitory effects (IC_50_: 58 nM)		(Yang et al., 2010)
	7-methoxycryptopleurine	*Tylophora indica*	potent coronavirus replication inhibitory effects (IC_50_: 20 nM)		(Yang et al., 2010)
	tetrandrine	*Stephania tetrandra*	potential antiviral activity against HCoV-OC43 infection (IC_50_: 14.51μM)		(Kim et al., 2019)
	fangchinoline	*Stephania tetrandra*	potential antiviral activity against HCoV-OC43 infection (IC_50_: 12.40μM)		(Kim et al., 2019)
	cepharanthine	*Stephania tetrandra*	potential antiviral activity against HCoV-OC43 infection IC_50_: 10.54μM)		(Kim et al., 2019)
	Homoharringtonine	*cephalotaxus hainanensis*	antiviral activity against diverse species of human and animal coronaviruses IC_50_ (12nM)		(Cao et al., 2015)

**Table 2:** Effective SARS-CoV-2 targets

Targets	Name	PDB code	Comparison with	The most potent inhibitors	Ref.
Viral attachment targets	Spike (S) protein to ACE2 receptor	2AJF:B	With other coronavirus species	-	(Ralph et al., 2020)
	S-ACE2		-	reduction of blood cholesterol levels leads to inhibition of the attachment of coronaviruses to host cells	(Baglivo et al., 2020)
Genome replication targets	Mpro (protease)	6lu7	Curcumin	Ribavirin….	(Kandeel and Al-Nazawi, 2020)
	RNA-dependent RNA polymerase (RdRp)	6NUR	Positive control (GTP, UTP)- negative control (Cinnamaldehyde, Thymoquinone)	Sofosbuvir, Ribavirin, and Remdisivir	
	Main protease	peptide-like: 6Y2F small molecules: 6W63	Crystal structures	Cobicistat, ritonavir, lopinavir, and darunavir	(Pant et al., 2020)
	RdRp; modeling	6NUR	Positive control (GTP, UTP)- negative control (Cinnamaldehyde, Thymoquinone) SARS-CoV-2 RdRp model and SARS HCoV RdRp (PDB ID: 6NUR) and hepatitis C virus (HCV) non-structural protein 5B (NS5B) RdRp (PDB ID: 2XI3)	Ribavirin, Remdesivir, Sofosbuvir, Galidesivir, and Tenofovir	(Elfiky, 2020)
	Main protease	5R7Y, 5R7Z, 5R80, 5R81, 5R82	-	A dock score of −6.5 or less is considered better	(Shah et al., 2020)
	Main protease-3CL^pro^	6LU7	SARS-C0V main protease (1UK4)	-	(Macchiagodena et al., 2020)
	SARS-CoV2 E (homolog to SARS-CoV E)	5X29	-	Belachinal, Macaflavanone E, and Vibsanol	(Gupta et al., 2020)

**Table 3:** The best docking results for the coronavirus main protease with drug-likeness properties

Compounds	Binding Energy (kcal/mol)	MW	Donor HB	Accept HB	QPlogPo/w	PSA	Type of compound
Fangchinoline	-10.1	608.733	1	8	5.585	64.094	Alkaloid
Hypericin	-9.7	504.452	4	6.5	2.132	156.274	Anthraquinone
Rutin	-9.6	610.524	9	20.55	-2.637	270.703	Glycosylated flavonoids
Swertifrancheside	-9.4	720.596	6.5	16.5	-1.597	134.231	Flavone-xanthone glucoside
Tingenone	-9.4	420.591	1	4.75	4.516	74.771	Pentacyclic triterpene
Amentoflavone	-9.3	538.466	4	7.5	2.81	194.19	Glycosylated flavonoids
Robustaflavone	-9.3	538.466	4	7.5	2.957	193.616	Glycosylated flavonoids
3-Isotheaflavin-3 gallate	-9.2	716.608	10	13.7	-0.157	263.627	Tannin
Hesperidin	-9.2	610.568	7	20.05	-1.324	239.922	Glycosylated flavonoids
Polyphyllin_I	-9.2	855.027	8	25.3	1.026	219.105	Triterpenoids
Sceptrin	-9.2	620.305	10	9	1.764	205.795	Marine metabolites
Soulattrolide	-9.2	404.462	1	5.7	4.221	68.472	Coumarin
Chebulagic acid	-9	954.672	13	26.15	-3.9	470.728	Benzopyran tannin
Savinin	-9	352.343	0	6	2.396	80.993	Lignan
Theaflavin-3, 3'digallate	-9	868.714	12	16.25	0.311	341.852	Tannin
Ursonic_acid	-9	454.692	1	4	6.176	62.974	Pentacyclic triterpenoid

**Table 4:** The best docking results for RNA-dependent RNA polymerase with drug-like properties

Compounds	Binding Energy (kcal/mol)	MW	Donor HB	Accpt HB	QPlog Po/w	PSA	Type of compound
**Chebulagic acid**	-10.5	954.672	13	26.15	-3.9	470.728	Benzopyran tannin
**Hesperidin**	-10.3	610.568	7	20.05	-1.324	239.922	Flavanone glycoside
**Suramin**	-10.1	1297.263	12	36	-0.965	508.544	Polysulphonated naphthylurea
**Eugeniin**	-10	938.672	15	22.95	-3.391	474.199	Ellagitannin
**Dipsacoside_B**	-9.5	1075.249	13	36	-3.04	331.507	Triterpenoids
**Amentoflavone**	-9.3	538.466	4	7.5	2.81	194.19	Glycosylated flavonoids
**Robustaflavone**	-9.2	538.466	4	7.5	2.957	193.616	Glycosylated flavonoids
**Sotetsuflavone**	-9.1	552.493	3	7.5	3.524	180.755	Biflavonoid
**Swertifrancheside**	-9.1	720.596	8	16.5	-0.182	295.835	Flavone-xanthone glucoside

**Table 5:** The best docking results for the spike protein with drug-like characteristics

Compounds	Binding Energy (kcal/mol)	MW	Donor HB	Accept HB	QPlog Po/w	PSA	Type of compound
**Suramin**	-9.7	1297.263	12	36	-0.965	508.544	Polysulphonated naphthylurea
**Amentoflavone**	-9.1	538.466	4	7.5	2.81	194.19	Glycosylated flavonoids
**Sotetsuflavone**	-8.5	552.493	3	7.5	3.524	180.755	Biflavonoid
**Chebulagic_acid**	-8.4	954.672	13	26.15	-3.9	470.728	Benzopyran tannin
**Robustaflavone**	-8.4	538.466	4	7.5	2.957	193.616	Glycosylated flavonoids
**Iguesterin**	-8.1	404.591	1	2.75	5.518	49.966	quinonoid triterpene
**Eugeniin**	-7.9	938.672	15	22.95	-3.391	474.199	Ellagitannin
**Polyphyllin_I**	-7.8	855.027	8	25.3	1.026	219.105	Triterpenoids
**Hypericin**	-7.7	504.452	4	6.5	2.132	156.274	Anthraquinone
**Verbascoside**	-7.7	624.594	9	20.3	-1.537	252.529	caffeoyl phenylethanoid glycoside
**3B-Friedelanol**	-7.6	428.740	1	1.7	7.124	17.623	Triterpenoids
**Astragaloside_A**	-7.6	784.980	9	21.9	0.484	207.345	Saponin
**Topsentin**	-7.6	342.356	3	3.250	3.285	99.250	Alkaloids

**Figure 7:**
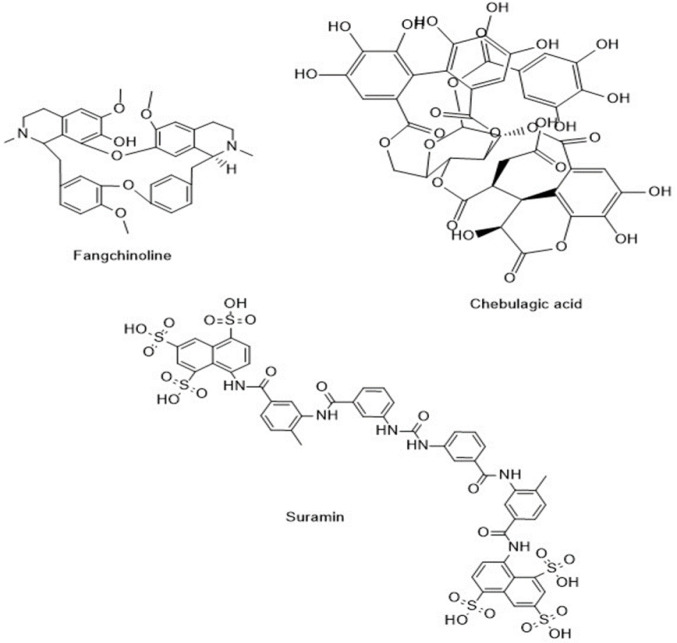
Chemical structure of fangchinoline, chebulagic acid and suramin

## Materials and Methods

### Preparation of a small-scale database (based on effective anti-viral natural products)

To find potential compounds effective in the treatment of coronavirus, an extensive search for articles published between 1990 and 2022 in electronic databases (including Google Scholar, Science Direct, PubMed and Scopus) based on the keywords coronavirus, COVID-19, SARS, MERS, Natural product, herb, plant and extract was done. Finally, effective natural compounds were selected and classified based on chemical structure. The desired compounds were drawn in SDF format and entered into the database. The molecular structure of each active compound was confirmed by literature mining and PubChem (https://pubchem.ncbi.nlm.nih.gov/). The selected natural compounds with anti-viral effects are listed in Table 1. 

### Protein preparation (selection effective SARS-CoV-2 targets)

For molecular docking study, the effective protein targets in SARS-CoV-2 should be identified first. In Table 2, the effective proteins are summarized along with the most effective combination and its identification code in the Protein Data Bank (PDB) (https://www.rcsb.org).

### Molecular docking process

Molecular docking is a computational method that aims to predict the molecular connection between small molecules (ligands) and macromolecular targets (desired receptors). The ability of the ligands for receptor inhibition is examined based on binding energy. The lower amount of ligand-receptor binding energy demonstrates the ability of the ligand for receptor inhibition. The AutoDock Vina software was utilized to conduct molecular docking studies on a designed small-scale database (Huey et al., 2012). The amount of binding energy is obtained based on kcal/mol. 

### Molecular dynamics study

Molecular dynamic simulation is an acceptable computer simulation method for simulating complex multi-particle systems by solving Hamilton's equations. In the molecular dynamic study, the dynamic behavior of proteins in the solvent is predicted by statistical mechanics (Hospital et al., 2015). In this study, the best-docked structures were subjected to molecular dynamics simulations. Molecular dynamics was calculated with GROMACS software.

### Prediction of drug-likeness properties

Drug-likeness determines qualitatively the chance of a small molecule to become an oral drug concerning bioavailability based on Lipinski's rule of five. In the drug-likeness study, the pharmacokinetic properties of a small molecule such as absorption, distribution, metabolism, and elimination were investigated (Di et al., 2009).

## Results

### Small-scale anti-viral natural products database

Some previous *in vitro* studies introduced effective natural compounds against other types of coronavirus infection. According to the literature, the effective natural compounds in previous studies were collected and classified in this study based on chemical structures. Different structures were classified as terpenoids, polyphenols, especially flavonoids and alkaloids. Table 1 summarizes the effective natural compounds for SARS-CoV-2 treatment. 

Terpenoids are an important class of naturally occurring organic chemicals with promising therapeutic effects against coronaviruses. In different studies, various types of terpenoids, such as saponin terpenoids (such as glycyrrhizin) (Cinatl et al., 2003), abietane terpenoids (Park et al., 2012b) and quinone methide terpenoids (Ryu et al., 2010b) have been effective against different types of coronaviruses. 

Polyphenols are a class of secondary metabolites that are found in many plants which include lignans, phenolic acids, flavonoids and stilbenes. Polyphenols and especially flavonoids, present potential anti-viral activity with safe administration and low toxicity (Kaul et al., 2021). Different types of polyphenols such as diarylheptanoids (such as curcumin) (Park et al., 2012a), flavonoids (such as lutein) (Yi et al., 2004) and chalcones (Park et al., 2016) were effective in previous coronavirus infections. 

Alkaloids are a large class of secondary metabolites with various therapeutic effects. Alkaloids have been considered potential anti-viral compounds against SARS-CoV-2. So far, various alkaloids have been investigated against coronaviruses and they have shown acceptable therapeutic effects (Majnooni et al., 2021). 

### Selection effective SARS-CoV-2 targets

The effective protein targets in SARS-CoV-2 should be identified and classified. The effective proteins and their identification code in the Protein Data Bank (PDB) (https://www.rcsb.org) are summarized in Table 2. Two major types of SARS-CoV-2 targets are (1) Proteins involved in SARS-CoV-2 attachment to host cells and (2) proteins involved in genome replication. (1) The first step in viral pathogenesis is the virus's attachment to host cells. The coronavirus glycoprotein is responsible for viral attachment. Spike (S) glycoprotein recognizes host cells and fuses with them. In β-coronaviruses, S glycoprotein binds to angiotensin-converting enzyme 2 (ACE2). Therefore, ACE2 is a potent receptor for the attachment of coronavirus (Li et al., 2003, Hoffmann et al., 2020). On the other hand, ACE2 expression increased during SARS-CoV-2 infection. Therefore, ACE2 inhibitors could be effective compounds in the treatment of COVID-19 (2). The most important enzymes involved in coronavirus genome replication are 3C-like protease (3CLpro) and papain-like protease (PLpro). Also, helicase and RNA-dependent RNA polymerase (RdRp) enzymes are essential for coronavirus multiplication. Inhibitors of these enzymes can play an effective role in treating COVID-19.

### Molecular docking

#### Docking of the small-scale designed database on the main protease of coronavirus

The crystal structure of the selected main protease of coronavirus (5R7Z) was downloaded from the PDB website. The final docking results on all the selected natural compounds are reported in supplementary data. In Table 3, the best Binding Energy results (X ≤ -9 kcal/mol) for the main protease are summarized.

#### Docking of the small-scale designed database on the RNA-dependent RNA polymerase (RdRp) of coronavirus

The three-dimensional crystal structure selected RNA-dependent RNA polymerase (6M71) was downloaded from the PDB site. The final docking results on all the selected natural compounds are reported in supplementary data. In Table 4, the best Binding Energy results (X ≤ -9 kcal/mol) for the main polymerase are summarized.

#### Docking of the small-scale designed database on the spike protein of coronavirus

The 3-D crystal structure of the coronavirus surface receptor (7BWJ) was downloaded from the PDB site. The final results of docking on all selected natural compounds are presented in supplementary data. The best results of Binding Energy (X ≤ -7.5 kcal/mol) for the Spike protein are summarized in Table 5.

### Molecular dynamics simulation

The molecular dynamic simulation was run with GROMACS package Version 2020.1, a high-performance Linux cluster (Abraham et al., 2015) to determine the behavior of selected best ligands in complex with main protease, RNA-dependent RNA polymerase and spike protein of SARS-CoV-2. 

### Molecular dynamics simulation of fangchinoline with the main protease

After the virtual screening, the best result was entered into the environment of water and ions for more detailed investigations. The stability of the fangchinoline (alkaloid structure) with the main protease complex was investigated with molecular dynamics stimulation (Figure 1).

Due to the presence of polar amino acids such as threonine, arginine, serine and glutamic acid in the active site of this enzyme, fangchinoline could bind strongly with about 3 to 4 hydrogen bonds at a distance of 0.35 nm from the active site. The drug-protein complex was stable and the deviation from the standard structure was about 0.3 nm. The Leonard Jones interactions (nonbinding interaction due to the presence of polar and charged groups) between fangchinoline and the virus main protease were about -180.65 kJ/mol. Molecular dynamics simulation showed that fangchinoline with its flexible structure could be able to create a ring in the active site of the protein, which leads to a stronger inhibitory effect in comparison to lopinavir (inhibitor of main protease). 

The calculated Gibbs free energy for the binding of fangchinoline with the main protease is about -42.26 kJ/mol, showing a strong inhibitory effect in the main protease in comparison to lopinavir (-30.9 kJ/mol) (Figure 2).

### Molecular dynamics simulation of chebulagic acid with RNA-dependent RNA polymerase (RdRp)

Based on virtual screening results, chebulagic acid with benzopyran tannin structure showed the best inhibitory effect on RdRp in the molecular docking study. Figure 3 shows the interaction between chebulagic acid and virus RdRp.

Molecular docking study and molecular dynamics simulation presented that the best antagonist of RdRp is remdesivir even in comparison to sofosbuvir and ribavirin. Virtual screening and molecular dynamic simulation showed that chebolagic acid binds to RdRp with a Gibbs free energy of -43.9 kJ/mol which is more potent than remdesivir with a Gibbs free energy of -28.8 kJ/mol.

In the investigation of the active site of this enzyme, it was observed that the location of remdesivir is slightly different from chebulagic acid. However, in the investigation of the ligand-protein interaction, it was observed that chebulagic acid is fitter in the active site. A powerful interaction between the phosphate and magnesium groups in the active site of the enzyme and ligands was observed (Figure 4).

### Molecular dynamics simulation of suramin with spike protein

Virtual screening on a small designed database revealed that suramin could strongly inhibit the spike protein. Figure 5 shows the interaction of suramin and spike protein of coronavirus.

Spike protein inhibitors are compounds that can interact strongly with the protein binding region (RBD) of this receptor and can compete with the receptors in the human body. Most of the compounds selected in this study had a high binding ability with this protein and were able to inhibit the binding level of this protein completely.

Studies have shown that polar amino acids play a role in establishing strong interactions with inhibitors through hydrogen bond formation. In spike protein, E484, N501, Q498, E406, D405, Q493, S494, R403, K417 and Y505 played the main role. The alpha-type (α variant) of spike protein has more transmission power than the original virus type. As shown in Figure 6-A, in this variant, asparagine at position 501 has been changed to tyrosine, which has reduced the binding power of suramin to spike protein. Of course, 13 other mutations have occurred in the α variant, but none of them are in the ACE2 binding site. In April 2021, another type of this virus was identified, which again had a key mutation in the active site of the spike protein. In the delta species, lysine at position 417 was changed to asparagine. Our studies showed that the selected compounds effective on the wild-type species could also be effective on the delta species (Figure 6B).

## Discussion

Among the natural compounds that were investigated in this study, three compounds fangchinoline, chebulagic acid and suramin were identified as the most effective in fighting SARS-CoV-2 infection. The structure of these compounds is shown in Figure 7.

Fangchinoline is an alkaloid compound with a bis-benzylisoquinoline structure which is isolated from the plant *Stephania tetrandra* of the Menispermaceae family, which is considered one of the traditional Chinese medicine plants, and its anti-cancer effects have been reported in many studies (Kim et al., 2019, Yang et al., 2020, Mérarchi et al., 2018). The binding energy of this compound in inhibiting the main protease of the coronavirus was -10.1 kcal/mol, which has shown the strongest inhibitory effect among the investigated natural compounds. Also, the investigation of drug-likeness properties for fangchinoline showed that this compound follows Lipinski's rule of five and could be orally active. Also, according to its polar surface area (PSA) level (64.094), fangchinoline could pass through the cell wall and penetrate the cell. Further molecular dynamics simulation demonstrated the high ability of fangchinoline to inhibit the main protease compared to lopinavir. Finally, fangchinoline placed powerfully in the active site of the main protease and inhibited this protein significantly compared to lopinavir. Despite the significant effects of fangchinoline in preclinical models, clinical and toxicological studies have not been conducted on this compound. 

Chebulagic acid is a type of benzopyran tannin found in plants of the genus *Terminalia*, especially *Terminalia chebula*, with the Persian name “*Halileh Zard*” (Chen and Li, 2006). Chebulagic acid is a potent antioxidant and has been reported to have various effects such as immune system suppression (Hamada et al., 1997), and liver protection (Kinoshita et al., 2007). In novel studies, chebulagic acid has been introduced as a promising compound with antiviral effects against influenza (Kandeel and Al-Nazawi, 2020, Duncan et al., 2020). Furthermore, an *in vitro* study showed that chebulagic acid could effectively inhibit 3CLpro (Du et al., 2021). The docking study revealed that chebulagic acid inhibited RdRp with a binding energy of -10.5 kcal/mol. The molecular dynamics simulation also confirmed the strong inhibitory effect of chebulagic acid compared to remdesivir. Although the analysis of drug-like properties is not appropriate, according to the structure of this compound, it should be mentioned that this compound is a hydrolyzable tannin, and *in silico* studies confirmed the effectiveness of hydrolysis compounds obtained from chebulagic acid (Duncan et al., 2020). *In vivo* toxicological studies have shown that chebulagic acid has minimal toxicity (Huang et al., 2012).

Suramin is a synthetic compound with a polysulphonated naphthylurea structure, which is used in the pharmaceutical market to treat Trypanosomiasis and prostate cancer by injection. Also, small doses of suramin have been used for autism treatment (Naviaux et al., 2017). Furthermore, an *in vitro* study confirmed the effectiveness of suramin in the prevention of coronavirus proliferation in human lung epithelial cells (Salgado-Benvindo et al., 2020). Suramin can inhibit spike protein in different mutations of coronavirus. Although suramin is not orally active, due to the clinical use of suramin by injection, it can be considered a valuable compound in the treatment of COVID-19. Suramin is a polypharmacological compound that targets multiple pathways. Despite its adverse effects, the diverse biological activities of suramin continue to be explored in both experimental and clinical studies (Wiedemar et al., 2020). 

Finally, the reported compounds have shown good effectiveness in the *in silico* study and can be used for future investigation for COVID-19 treatment. 
